# Gastrointestinal Symptoms in HIV-Infected Patients: Female Sex and Smoking as Risk Factors in an Outpatient Cohort in Brazil

**DOI:** 10.1371/journal.pone.0164774

**Published:** 2016-10-17

**Authors:** Annelisa Silva e Alves de Carvalho Santos, Erika Aparecida Silveira, Marianne de Oliveira Falco

**Affiliations:** Post-Graduation Program in Health Sciences, Faculty of Medicine, Federal University of Goiás, Goiânia, Goiás, Brazil; National and Kapodistrian University of Athens, GREECE

## Abstract

This study aimed to estimate the incidence of gastrointestinal symptoms (GIS) and associated factors in an outpatient cohort of people living with HIV/AIDS (PLWHA) followed between October 2009 and July 2011. We evaluated nausea and/or vomiting, dyspepsia, heartburn, diarrhea, constipation, and flatulence. The outcome variable was the presence of three or more GIS. Sociodemographic (sex, skin color, age, income, years of schooling), lifestyle (smoking status, alcohol consumption, physical activity level), clinical (antiretroviral therapy, time of HIV infection, CD4 lymphocyte count, viral load), and anthropometric (nutritional status and waist circumference) variables were investigated. Data on sociodemographic and lifestyle variables were collected through a pre-tested and standardized questionnaire. CD4 count was determined by flow cytometry and viral load by branched DNA (bDNA) assays for HIV-1. All variables were analyzed at a p<0.05 significance level. Among 290 patients, the incidence of three or more GIS was 28.8% (95% CI 23.17 to 33.84) and 74.48% presented at least one symptom. Female gender (IR 2.29, 95% CI 1.63 to 3.22) and smoking status (IR 1.93, 95% CI 1.30 to 2.88) were risk factors for the presence of three or more GIS after multivariate Poisson regression. A high incidence of gastrointestinal symptoms was found among PLWHA, and it was significantly associated with female sex and tobacco use. Those results reinforce the relevance of investigating the presence of GIS in PLWHA as it may affect treatment adherence.

## Introduction

Despite the decline in the number of new cases of HIV infection in recent years, the number of people living with HIV/AIDS (PLWHA) is increasing. In a global perspective, the number of individuals living with the disease now exceeds 35 million [1]. In Brazil, there are currently 718,000 PLWHA, and in the last ten years, 37,446 cases on average were reported per year [2].

The increasing number of PLWHA is mainly due to the use of antiretroviral drugs to treat HIV infection, contributing to a significant reduction in morbidity and mortality related to HIV. The disease then becomes a chronic condition with new perspectives for management and treatment [3,4]. At the same pace, new challenges emerge to be addressed. Although antiretroviral therapy (ART) has contributed substantially to increase PLWHA life expectancy, short and long-term adverse effects may compromise adherence to drug treatment and impair quality of life [5,6].

Gastrointestinal symptoms (GIS) are among the short-term adverse effects of ART, usually transient, with resolution within a few months of ART use. Additionally, adjuvant therapies can be employed in order to alleviate the undesirable effects of the medication [7]. Some strategies for GIS management include modification of dietary habits and medication prescription for symptom relief [5,8–11].

GIS may also be a result of HIV infection itself [12,13]. Depletion of immune cells leads to increased susceptibility to opportunistic infections in the gastrointestinal tract of HIV-infected individuals, damaging the gastrointestinal mucosa [14,15]. Other causes for the emergence of gastrointestinal symptoms may be related to nutritional status and sex [16].

Gastrointestinal abnormalities in PLWHA have been reported since the advent of AIDS [17]. Among them, GIS are the most frequent complaints, especially diarrhea, nausea, and vomiting. However, studies on the incidence of these symptoms and associated factors in adults with HIV/AIDS are scarce in the literature [18,19]. Thus, this paper aims to estimate the incidence of GIS in PLWHA—including nausea and/or vomiting, dyspepsia, heartburn, diarrhea, constipation and flatulence—and to identify the associated factors with the presence of three or more of these symptoms.

## Materials and Methods

This research is part of a larger study called "Predictors of coronary disease and evaluation of metabolic parameters in a cohort of adult patients with HIV/AIDS"–PRECOR [20,21]. The study was conducted at the Outpatient Clinic of Infectious and Parasitic Diseases (OCIPD) of the Hospital das Clínicas of the Federal University of Goiás in Goiânia, Brazil. Data collection occurred between October 2009 and July 2011, which was the period of follow-up of PRECOR. However, the outcome variable of the present study (the presence of three or more gastrointestinal symptoms) was evaluated as incident in the last week before the interview, characterizing an acute form of ART-related gastrointestinal symptoms. Inclusion criteria were age ≥ 19 years and HIV infection, regardless ART use. Pregnant, and lactating women and those with an opportunistic infection diagnosed less than two months before recruitment or longer, but without clinical resolution within that period, were excluded from the study sample.

### Recruitment and logistics of data collection

The infectologist physician invited the patients who met eligibility criteria to participate in the study by the time of the regular medical consult in OCIPD and assigned those who agreed to participate to the responsible researchers. A cardiologist, nutritionists, and undergraduates composed the research group. Data collection on sociodemographic, clinical and smoking status variables occurred during the interview with the cardiologist. Subsequently, the cardiologist referred the patients for a consult with a nutritionist, who applied a questionnaire on the incidence of gastrointestinal symptoms, alcohol consumption, and physical activity level. The research team developed the questionnaire used for data collection, except for smoking status, alcohol consumption, and physical activity level assessment. The questionnaire was standardized and previously tested before the study commencement. Finally, a trained anthropometrist performed a physical assessment of the patients. Out of 337 patients included in the larger study, 290 patients were assigned to a nutritional consultant and they formed the sample of this study.

We investigated sociodemographic, lifestyle, clinical and anthropometric variables as potential associated factors for the presence of three or more GIS. Detailed information on data collection is described below.

### Gastrointestinal symptoms

The questionnaire on gastrointestinal symptoms comprised questions about experiencing nausea and/or vomiting, dyspepsia, heartburn, diarrhea, constipation and flatulence in the previous week of the interview, characterizing an acute form of gastrointestinal symptoms The Rome III criteria were used for the definition of the investigated symptoms. We considered the presence of three or more GIS as the outcome variable for statistical analysis. The incidence of at least one gastrointestinal symptom and the number of experienced symptoms were also investigated.

### Sociodemographic and lifestyle data collection

Sociodemographic (gender, skin color, age, income and years of schooling) and lifestyle variables (smoking status, risk consumption of alcohol and physical activity level) were assessed through a pre-tested and standardized questionnaire.

Income was clustered into tertiles. The first tertile was income up to US$ 243; the second tertile was revenue from US$ 244 to US$ 450, and the third income tertile was equal or higher than US$ 451. Smoking status and alcohol consumption were investigated according to the Pan American Health Organization (OPAS—Organización Panamericana de la Salud) [22]. Smoker was defined as one who currently smoke or had quit smoking for less than six months; former smoker who had quit smoking for more than six months; and nonsmoker who had never smoked. Alcohol consumption was assessed as risk consumption, i.e., 30 g of ethanol/day for men and 15 g ethanol/day for women [23].

The short version of the International Physical Activity Questionnaire (IPAQ) was used to assess physical activity level [24]. Moderate and vigorous physical activity level categories were clustered in the analysis, thus classifying individuals as active or sedentary regardless the intensity of physical activity level.

### Clinical examination and anthropometric measurements

Clinical characteristics were time since diagnosis of HIV infection, ART use, duration of ART use, class of antiretroviral drug (nucleoside reverse-transcriptase inhibitor (NRTI), non-nucleoside reverse transcriptase inhibitor (NNRTI) and protease inhibitor (PI)), CD4 T + lymphocytes count, and viral load. Anthropometric variables were waist circumference (WC) and nutritional status (Body Mass Index—BMI).

ART use was assessed as general use (yes or no) and categorized by antiretroviral classes (NRTI, NNRTI, and PI). The time since HIV diagnosis and the time of ART use were calculated based on the difference in years from the date of data collection and both events dates informed by the patient. Information on CD4 count and viral load were obtained from the patient’s medical chart, as these tests are performed routinely in the reference outpatient clinic. CD4 count was determined by flux cytometry and viral load by branched DNA (bDNA) assays for HIV-1 (Versante HIV-1 RNA 3.0 assay). CD4+ lymphocyte count (cells/mm^3^) was classified as ≤350 and >350 [25] and viral load (copies/ml) as <50 and ≥ 50.

The anthropometric measurements were performed according to standardized procedures [26]. To measure body weight, a digital scale with 150 kg capacity and 100 g accuracy was used (Tanita BC-558 Ironman). Height was measured to the nearest 0.1 cm with a 150 cm length non-elastic tape, at 50 cm from the ground, affixed to a wall without a baseboard. The patients were instructed to be barefooted during weight and height measurement. BMI was calculated as weight (kg) divided by the square of height (m^2^). Nutritional status was classified as: 1) underweight / normal weight (BMI <18.5 kg/m^2^ and between 18.5–24.9 kg/m^2^); 2) overweight (BMI between 25.0 to 29.9 kg/m^2^); and 3) obesity (BMI ≥ 30.0 kg/m^2^) [27]. Waist circumference (WC) was measured at the largest extension of the abdomen in a horizontal plane with a non-elastic measuring tape. WC was categorized as normal when <80 cm in women and <94 cm in males; increased when ≥ 80 and <88 cm in females and between ≥ 94 and <102 cm in males; and greatly increased when ≥ 88 cm in women and ≥ 102 cm in males [27].

### Sample size

Sample size calculation was performed with a posteriori data of our research using Epi Info 7.0 software with a confidence level of 95% and 80% power. An unexposed to exposed ratio of 1.56 and 1.14 for the variables sex and smoking resulted in a sample size of 109 and 195 patients, respectively. Thus, the number of patients was sufficient to detect differences in the analysis of the outcome of interest.

### Statistical analysis

Statistical analysis was carried out in Stata/SE statistical package version 12.0 [28]. Absolute and relative frequencies of GIS were computed. Incidence proportion, incidence ratio (IR) and 95% confidence interval (CI) of the presence of three or more GIS according to independent variables were estimated. Wald’s test was used to test the statistical significance of associations, considering a p-value <0.05 as statistically significant. The Chi-square test was employed to test if there was a linear trend for the number of gastrointestinal symptoms by ART use. Variables with p-value ≤0.20 in bivariate analyses were included in a multivariate Poisson regression model with hierarchical analysis.

### Ethics

The Hospital das Clínicas of the Federal University of Goiás Ethics Committee approved the larger study (protocol number 006/2009) and all patients who agreed to participate signed a consent-to-disclose form.

## Results

The incidence of at least one GIS was 74.48% (95% CI 69.06 to 79.40), and 28.28% of patients had three or more GIS (95% CI: 23.17 to 33.84). A significant association with GIS and use of ART was not observed (Table 1).

**Table 1:** Incidence of gastrointestinal symptoms according to antiretroviral therapy use in HIV-infected adult patients.

		Incidence	Incidence		p-value^a^
			On ART	ART naïve	
		n (%; 95% CI)	n (%)	n (%)	
At least one symptom		216 (74.48; 69.06–79.40)	150 (75.76)	66 (71.74)	0.479
Three or more symptoms		82 (28.28; 23.17–33.84)	58 (29.29)	24 (26.09)	0.577
Number of symptoms^b^					
	None	74 (25.52; 20,60–30.94)	48 (24.24)	26 (28.26)	-
	One	63 (21.72; 17,12–26.92)	46 (23.23)	17 (18.48)	0.302
	Two	71 (24.48; 19,64–29.85)	46 (23.23)	25 (27.17)	0.992
	Three	43 (14.83; 10,94–19.45)	34 (17.17)	9 (9.78)	0.092
	Four or more	39 (13.45; 9.74–17.92)	24 (12.12)	15 (16.30)	0.727

CI, confidence interval; ART, antiretroviral therapy.

^a^Wald’s test.

^b^χ^2^ linear trend: p = 0.818.

Regarding sociodemographic and lifestyle characteristics, the presence of three or more GIS was associated with female sex (IR: 2.26, 95% CI: 1.60–3.20); first (IR: 2.00, 95% CI: 1.23–3.24) and second tertiles of income (IR: 1.72, 95% CI: 1.03–2.87); and smoking status (IR: 1.90, 95% CI: 1.26–2.86) (Table 2).

**Table 2:** Incidence and incidence ratio of three or more gastrointestinal symptoms according to sociodemographic and lifestyle characteristics of HIV-infected patients.

		n (%)	Three or more gastrointestinal symptoms		
			Incidence	IR	p-value^a^
			n (%)	(95% CI)	
Sex					0.000
	Male	226 (77.93)	50 (22.12)	1.00	
	Female	64 (22.07)	32 (50.00)	2.26 (1.60–3.20)	
Skin color					0.684
	White	147 (50.69)	40 (27.21)	1.00	
	Brown/Black	143 (49.31)	42 (29.37)	1.08 (0.75–1.56)	
Age					0.970
	19–29	78 (26.90)	21 (26.92)	1.00	
	30–39	99 (34.14)	28 (28.28)	1.05 (0.65–1.70)	
	40–49	76 (26.21)	23 (30.26)	1.12 (0.68–1.86)	
	50 or more	37 (12.76)	10 (27.03)	1.00 (0.53–1.91)	
Income					0.020
	1^st^ tertile	96 (33.22)	35 (36.36)	2.00 (1.23–3.24)	
	2^nd^ tertile	89 (30.80)	28 (31.46)	1.72 (1.03–2.87)	
	3^rd^ tertile	104 (35.99)	19 (18.27)	1.00	
Years of schooling					0.468
	Up to 4 years	40 (13.79)	14 (35.00)	1.43 (0.84–2.43)	
	5–8 years	57 (19.66)	19 (33.33)	1.36 (0.83–2.21)	
	9–11 years	114 (39.31)	28 (24.56)	1.00	
	12 years or more	79 (27.24)	21 (26.58)	1.08 (0.66–1.76)	
Smoking status					0.009
	Nonsmoker	151 (52.07)	33 (21.85)	1.00	
	Smoker	70 (24.14)	29 (41.43)	1.90 (1.26–2.86)	
	Former smoker	69 (23.79)	20 (28.99)	1.33 (0.82–2.14)	
Risk consumption of alcohol					0.942
	No	28 (28.57)	7 (25.00)	1.00	
	Yes	70 (71.43)	18 (25.71)	1.03 (0.48–2.20)	
Physical activity level					0.715
	Active	115 (39.79)	48 (27.59)	1.00	
	Sedentary	174 (60.21)	34 (29.57)	0.93 (0.64–1.35)	

IR, incidence ratio; CI, confidence interval.

^a^Wald’s test.

Most patients (39.57%) presented more than three years of ART use while about 35% were on ART for less than a year. Nevertheless, the presence of three or more GIS was not significantly associated with the time of ART use, as expected. All patients were prescribed NRTI in their antiretroviral regimen. The use of NRTI was not statistically associated with the presence of three or more GIS (data not shown in tables). The use of NNRTI was a protective factor for the incidence of three or more GIS (IR: 0.59, 95% CI: 0.39–0.89). The use of IP (IR: 1.56, 95% CI: 1, 03 to 2.38) and greatly increased WC (IR: 2.21, 95% CI: 1.51 to 3.25) were risk factors for the outcome variable (Table 3).

**Table 3:** Incidence and incidence ratio of three or more gastrointestinal symptoms according to clinic and anthropometric characteristics of HIV-infected patients.

		n (%)	Three or more gastrointestinal symptoms		
			Incidence	IR	p-value^a^
			n (%)	(95% CI)	
NNRTI					0.013
	No	45 (22.28)	20 (44.44)	1.00	
	Yes	157 (77.72)	41 (26.11)	0.59 (0.39–0.89)	
PI					0.036
	No	147 (73.50)	39 (26.53)	1.00	
	Yes	53 (26.50)	22 (41.51)	1.56 (1.03–2.38)	
Time of HIV infection					0.732
	< 1 year	72 (26.47)	18 (25.00)	1.00	
	1–3 years	84 (30.88)	25 (29.76)	1.19 (0.71–2.00)	
	> 3 years	116 (42.65)	35 (30.17)	1.21 (0.74–1.96)	
Time of ART use					0.613
	< 1 year	66 (35.29)	20 (30.30)	1.25 (0.72–2.15)	
	1–3 year	47 (25.13)	15 (31.91)	1.31 (0.73–2.35)	
	> 3 year	74 (39.57)	18 (24.32)	1.00	
T CD4+ lymphocyte count					0.282
	≤ 350 cells/mm^3^	76 (27.14)	18 (23.68)	1.00	
	> 350 cells/mm^3^	204 (72.86)	62 (30.39)	1.28 (0.81–2.02)	
Viral load					0.157
	< 50 copies/ml	153 (55.23)	49 (32.03)	1.00	
	≥ 50 copies/ml	124 (44.77)	30 (24.19)	0.75 (0.51–1.11)	
Nutritional Status					0.132
	Underweight / Normal weight	179 (66.30)	44 (24.58)	1.00	
	Overweight	78 (28.89)	24 (30.77)	1.25 (0.82–1.91)	
	Obesity	13 (4.81)	6 (46.15)	1.88 (0.99–3.57)	
Waist circumference					0.000
	Normal	166 (61.48)	40 (24.10)	1.00	
	Increased	59 (21.85)	10 (16.95)	0.70 (0.38–1.32)	
	Greatly increased	45 (16.67)	24 (53.33)	2.21 (1.51–3.25)	

IR, incidence ratio; CI, confidence interval; NNRTI, non-nucleoside reverse transcriptase inhibitor; PI, protease inhibitor; ART, antiretroviral therapy.

^a^Wald’s test.

In multivariate analysis, the following variables were included as hierarchical levels. In the first level was gender and income; in second was smoking; in third was the use of NNRTI, PI use, and viral load; and in the fourth level was nutritional status and WC. After adjusting the model, the variables that remained significantly associated with the presence of three or more GIS were female gender and smoking status (Table 4).

**Table 4:** Multivariate analysis by Poisson regression for associated factors with the presence of three or more gastrointestinal symptoms in HIV-infected patients.

		Three or more gastrointestinal symptoms	
		_a_IR (95% CI)	p-value^a^
Sex			
	Male	1.00	-
	Female	2.29 (1.63–3.22)	0.000
Smoking status			
	Nonsmoker	1.00	-
	Smoker	1.93 (1.30–2.88)	0.001
	Former smoker	1.29 (0.81–2.04)	0.276

_a_IR, adjusted incidence ratio; CI, confidence interval.

^a^Wald Test.

## Discussion

This study provides a significant contribution to estimating the incidence of gastrointestinal symptoms in PLWHA. It is noteworthy that, in a broad review of the literature, studies that evaluated the presence of dyspepsia, heartburn, constipation and flatulence along with nausea and/or vomiting and diarrhea were not found. Thus, this study is pioneering to evaluate those six symptoms simultaneously. High incidence of GIS in PLWHA was identified. Studies that evaluated GIS in grouped form were not found in the literature review, becoming difficult to compare the incidence of symptoms with other research.

Our research has also identified female gender and smoking status as risk factors for the presence of three or more GIS. Once again, no investigations on risk factors for the presence of gastrointestinal symptoms in PLWHA were found, which is another important contribution of this study. A comparative study on incidences of gastrointestinal symptoms in patients with ART regimen, consisting of two NRTIs and one PI, found the incidence of diarrhea varying between 4% and 19%; nausea between 2% and 9%; and the incidence of vomiting between 2% and 5%. Possibly, these symptoms were adverse effects of the antiretroviral drugs under investigation [18].

In a cohort of HIV-positive patients, Knox et al. found a prevalence of 38.9% of at least one diarrhea episode in the previous month of the interview. The prevalence of diarrhea was significantly higher in men than in women, contrasting with the results found in our study [19]. A review about gender influence on gastrointestinal physiology revealed that are differences between men’s and women’s gastrointestinal tract, although some aspects remain unclear. The main difference is related to intestinal motility, which tends to be slower in women. The levels of steroid hormones vary according to the physiological state (i.e., pre and post menopause, pregnancy), influencing the intestinal motility [29]. In another point of view, some studies indicate that men are less likely to refer health issues than women [30, 31]. This theory can explain why gastrointestinal symptoms report was higher among women than men in our study.

The prevalence of gastrointestinal symptoms in population-based studies is variable. Most research in the literature reports the prevalence of functional gastrointestinal disorders, related or not to other gastrointestinal diseases as gastroesophageal reflux disease [32,33] and the investigation of associated factors [16,34]. There is no consensus on the prevalence of gastrointestinal symptoms in the general population. A systematic review of 10 studies has identified the prevalence of upper gastrointestinal symptoms varying from 8% to 54%. Such variation is mainly due to the different criteria employed in defining the symptoms. The broader the definition used, the higher the prevalence of symptoms [35].

The association between smoking and gastrointestinal disorders has been widely reported in the literature, mostly evidencing the role of nicotine toxicity and/or its byproducts in the development of gastrointestinal diseases such as ulcers, cancers, Crohn’s disease and gastroesophageal reflux [36–38]. Nicotine stimulates parasympathetic autonomic system, increasing muscular tonus and gastrointestinal motility [39]. This mechanism is a possible explanation for the association between tobacco use and gastrointestinal symptoms found in the present study, maintained in the multivariate analysis.

Typical ART regimens usually include two NRTI as main support and one PI or NNRTI, although there are variations. With the facilitated access to antiretroviral therapy, more patients have long exposition time to the treatment, naturally resulting in higher toxicity report. Gastrointestinal symptoms are the most common complaints among patients on ART, primarily in its first year of use, and can be associated with all antiretroviral classes [40]. Regarding NRTIs, studies report GIS related to emtricitabine, didanosine, tenofovir and zidovudine; among NNRTIs, the symptoms can be present with the use of etravirine, primarily nausea; and PIs, which are more commonly associated with GIS, ritonavir, and nelfinavir [7,41]. Nevertheless, most studies that evaluate new antiretrovirals efficacy and safety compare the intervention group with a conventional treatment group, i.e., patients on antiretroviral use. Thus, the observation of gastrointestinal symptoms supposedly related to the new drug on test could be covert by the HIV infection itself, once the incidence and/or prevalence of gastrointestinal symptoms before ART commencement are usually not investigated.

Significant association between three or more gastrointestinal symptoms and ART use was not observed in our study, confirming that gastrointestinal symptoms can be present either in patients on ART and in those who are not. Therefore, it can be stated that gastrointestinal symptoms arise not only from ART use but also from HIV infection natural disease history. Additionally, other issues can be involved in GIS origin. HIV-infected patients suffer a high emotional burden due to the disease itself and the stigmatization of people living with HIV/AIDS, which remains nowadays and could trigger the emergence of GIS [42]. Another hypothesis is that GIS could be related to diseases carried by food and water, considering that PLWHA has more susceptibility to infections. These issues must be approached in future research to contribute to the elucidation of the mechanisms involved in this process.

PLWHA with low CD4 + T cells count are more susceptible to opportunistic infections associated with the presence of gastrointestinal symptoms, either in the upper and lower gastrointestinal tract [14,15,43]. In the present study, approximately 25% of patients had CD4 <350 cells/mm^3^ and no association between three or more gastrointestinal symptoms and low CD4 lymphocyte count was observed. One factor that could explain the lack of association between these variables is the exclusion of patients diagnosed with an opportunistic infection in the larger study.

A recent study with 384 PLWHA on ART examined the impact that adverse effects exert on social life. PLWHA perceived that other people avoid contact with them due to adverse effects of ART, such as nausea, vomiting, loss of appetite and diarrhea, and the otherwise is true. That is, PLWHA experiencing these symptoms also avoid social life due to these adverse effects [44]. These findings reinforce the importance of research on GIS in PLWHA for its appropriate treatment, with consequent improvement of the quality of life and HIV/AIDS care.

This research addresses an important issue that is rarely evaluated in a comprehensive manner as here presented. High incidence of gastrointestinal symptoms in PLWHA and its association with female sex and smoking status was observed. Thus we recommend the screening of gastrointestinal symptoms especially among women and smokers in routine HIV/AIDS centers care. Additionally, as smoking was a risk factor for gastrointestinal symptoms in our study, we also recommend tobacco cessation to prevent and/or attenuate the emergence of GIS.

Considering that the high incidence of gastrointestinal symptoms may affect the HIV/AIDS treatment, increasing the treatment dropout rate and decreasing the level of adherence to drug therapy and other interventions, as well as affecting the quality of life, especially social life, it is important to study the influence of gastrointestinal symptoms on these issues not addressed in our study.
